# COVID-19 pandemic and therapy with ibuprofen or renin-angiotensin system blockers: no need for interruptions or changes in ongoing chronic treatments

**DOI:** 10.1007/s00210-020-01890-6

**Published:** 2020-05-15

**Authors:** Oliver Zolk, Susanne Hafner, Christoph Q. Schmidt, on behalf of the German Society for Experimental and Clinical Pharmacology and Toxicology (DGPT)

**Affiliations:** 1 Institute of Clinical Pharmacology, Brandenburg Medical School Theodor Fontane, Immanuel Klinik, Seebad 82/83, Rüdersdorf, 15562 Germany; 2 Institute of Pharmacology of Natural Products and Clinical Pharmacology, Ulm University, 89081 Ulm, Germany

**Keywords:** COVID-19, SARS-CoV-2, Angiotensin-converting enzyme inhibitors, Angiotensin receptor antagonists, Non-steroidal anti-inflammatory agents

## Abstract

Scientists hypothesized that drugs such as ibuprofen or renin-angiotensin system (RAS) blockers could exacerbate the novel coronavirus disease COVID-19 by upregulating the angiotensin-converting enzyme 2 (ACE2), which serves as an entry receptor for the coronavirus SARS-CoV-2. This hypothesis was taken up by the lay press and led to concerns among doctors and patients whether the use of these drugs was still safe and justified against the background of the pandemic spread of SARS-CoV-2 with an increasing number of cases and deaths. In this article, we summarize what is known about the effect of RAS blockers or non-steroidal anti-inflammatory drugs (NSAIDs) on the course of COVID-19 disease. In the case of RAS inhibition, we also find evidence for the opposite hypothesis, namely, that RAS inhibition in COVID-19 could be protective. In view of the inconsistent and limited evidence and after weighing up the benefits and risks, we would not currently recommend discontinuing or switching an effective treatment with RAS blockers. NSAIDs should be used at the lowest effective dose for the shortest possible period. The choice of drug to treat COVID-19-associated fever or pain should be based on a benefit-risk assessment for known side effects (e.g., kidney damage, gastrointestinal ulceration).

Recent media reports and publications question whether some medicines could worsen the novel coronavirus disease COVID-19 that is caused by infection with the SARS-CoV-2 coronavirus. These reports mention angiotensin-converting enzyme (ACE) inhibitors and angiotensin receptor blockers (ARBs), which are commonly used drugs to treat patients with hypertension, heart failure, or kidney complications in diabetes mellitus, as well as ibuprofen, which is an over-the-counter drug prescribed or used to treat arthritis and rheumatic diseases among other indications. This article summarizes current evidence regarding the risk of these drugs during an unfavorable course of COVID-19.

## Epidemiological data

The most common comorbidities reported in epidemiological studies of patients with COVID-19 are hypertension, diabetes mellitus, coronary heart disease, and cerebrovascular disease. A higher number of comorbidities, which increase with age, are correlated with poorer clinical outcomes (Guan et al. 2020; Yang et al. 2020; Zhang et al. 2020). Epidemiological data show that the probability of dying from an infection with the SARS-CoV-2 virus increases with age, from 0.2 in patients ≤ 39 years old to 8% in patients aged 70–79 years and 15% in patients ≥ 80 years old (The Novel Coronavirus Pneumonia Emergency Response Epidemiology Team 2020; World Health Organization 2020). Therefore, it is not surprising to find an association between the use of drugs that are more commonly prescribed in older patients and poorer outcomes of COVID-19. However, this association alone does not yet describe anything about causality.

## Role of angiotensin-converting enzyme 2 (ACE2) in COVID-19

The reason for suspicion that some drugs might worsen the prognosis of COVID-19 stems from current reports that, for example, ACE inhibitors may lead to an increase in the number of ligands to which the SARS-CoV-2 coronavirus docks. This hypothesis was expressed in a commentary in *Nature Reviews Cardiology* (Zheng et al. 2020) and a letter in *The British Medical Journal* (Sommerstein and Gräni 2020). The angiotensin-converting enzyme 2 (ACE2), a membrane-bound aminopeptidase, serves as a receptor for the coronavirus and therefore an entry portal into cells. ACE2 is also the entry receptor for SARS-CoV, which caused the 2002–2004 SARS outbreak. During therapy with drugs, such as ARBs or ACE inhibitors, more ACE2 receptors are formed, theoretically producing more docking sites for the new coronavirus (Zheng et al. 2020). Because an ACE inhibitor (lisinopril) and an ARB (losartan) are both among the 10 most commonly used drugs with a combined 152 million prescriptions per year in the USA alone (IQVIA Institute 2019), these patients would represent a substantial group of people at risk.

Among the coronaviral structural proteins, the spike (S) protein facilitates viral entry into the host cell. The S protein consists of two functional domains, the S1 domain and the S2 domain. The S1 domain mediates the attachment of the virus to surface receptors of the host cell, whereas the S2 domain mediates subsequent fusion between viral and host cell membranes and endocytic entry of the virus. Hoffmann et al. have recently shown that the SARS-CoV-2 S protein engages ACE2 as the crucial entry receptor and employs the host cell serine protease TMPRSS2 for S protein priming (Hoffmann et al. 2020). Furthermore, the direct binding of the SARS-CoV-2 S protein to ACE2 has been confirmed by recent cryo-EM studies (Walls et al. 2020).

ACE2 is primarily located in the lungs, where it is mostly expressed in alveolar epithelial type II cells. Type II cells secrete surfactant and hence are critical to the gas exchange function of the lungs. Injury to these cells could explain the severe lung injury observed in COVID-19 pneumonia. ACE2 is also found in the heart, intestines, kidneys, and blood vessels and in soluble form in serum, which may explain why COVID-19, in addition to pneumonia, can cause multiple organ failure in severe cases.

## ACE2 as part of the renin-angiotensin system and its role in lung injury

ACE2 constitutes an important alternative renin-angiotensin system (RAS) pathway that counterbalances the classical RAS pathway (Guignabert et al. 2018). In the classical RAS pathway, angiotensin II, which is formed from angiotensin I by ACE1, induces aldosterone secretion, salt and water retention, inflammation, and severe arteriolar vasoconstriction via binding to the AT1 receptor. In contrast, ACE2 primarily hydrolyzes angiotensin II into two other biologically active products of the RAS cascade, angiotensin-(1-9) and angiotensin-(1-7), which promote vasodilation and also reduce cell growth and inflammatory responses (Guignabert et al. 2018).

Animal experiments in response to the 2002–2004 SARS outbreak have shown that infection with SARS-CoV leads to dysregulation of RAS with reduced ACE2 expression and an increase in angiotensin II, which increases lung damage (Kuba et al. 2005). Notably, the injection of SARS-CoV S protein into mice worsened acute lung failure in vivo, but this effect could be attenuated by blocking the classical RAS pathway (Kuba et al. 2005). Imai et al. observed that ACE2 protected against severe acute lung injury in an experimental murine model of acute lung injury, whereas components of the classical RAS pathway, including ACE1, angiotensin II, and the AT1A receptor, promoted disease pathogenesis, induced lung edema, and impaired lung function. Furthermore, recombinant ACE2 protected the mice from severe acute lung injury (Imai et al. 2005).

Current evidence therefore suggests that ACE2 serves both as an entry receptor of SARS-CoV-2 and to protect the lungs from injury, which may be the case with COVID-19. Interestingly, Monteil et al. showed that human recombinant soluble ACE2 (hrsACE2), which has already been tested in phase 1 and phase 2 clinical trials for the treatment of acute lung injury and pulmonary hypertension, can significantly block the early stages of SARS-CoV-2 infection in vitro (Monteil et al. 2020). As a result, a phase 2 clinical trial with hrsACE2 for the treatment of COVID-19 was recently initiated in Europe (EudraCT Number: 2020-001172-15).

## Effects of drugs on ACE2 expression

Our knowledge about ACE2 regulation in response to drugs is predominantly based on animal experiments. In rodents, treatment with ACE inhibitors or ARBs increased cardiac or renal ACE2 expression and/or ACE2 activity (Ferrario et al. 2005; Soler et al. 2009). In animal models of arterial hypertension, myocardial infarction, pressure overload, or diabetes mellitus, cardiac ACE2 was downregulated compared with healthy control animals (Ocaranza et al. 2006; Tikellis et al. 2006; Qiao et al. 2015; Wang et al. 2016; Zhao et al. 2019). Treatment with an ARB, ibuprofen, or pioglitazone reversed the decrease in ACE2 expression in diseased animals, whereas almost no effect on ACE2 expression was observed in control animals (Qiao et al. 2015; Wang et al. 2016; Zhao et al. 2019). Other COX-2 inhibitors and PPAR-γ ligand have not been investigated, and the precise mechanisms were not established. Thus, it remains unclear whether effects of ibuprofen and pioglitazone on ACE2 expression are drug class effects.

Data in humans are scarce. For most patients with heart failure and during the acute phase of myocardial infarction, treatment with RAS inhibitors showed an increase in cardiac or circulating ACE2, which was explained by the severity of heart disease rather than RAS inhibitor treatment (Zisman et al. 2003; Goulter et al. 2004; Epelman et al. 2009). Although animal data suggest that drugs such as ARBs, ACE inhibitors, ibuprofen, or pioglitazone can upregulate cardiac or renal ACE2, the evidence from animal experiments is not fully consistent, and it remains unclear whether the results are transferable to humans. Although the primary target of SARS-CoV-2 is ACE2 on the cell surface of alveolar type II cells, data on drug-induced changes in pulmonary ACE2 are lacking. Although there is evidence that comorbidities can have significant effects on ACE2 expression, it is difficult to assess the net effect on ACE2 expression in patients. Moreover, it would have to be shown that a drug-induced increase in ACE2 expression is sufficient to facilitate SARS-CoV-2 entry.

## Risks related to the use of NSAIDs in COVID-19

The local inflammatory process in severe COVID-19 can induce parenchymal injuries, such as damage to the alveolar-capillary unit (Shi et al. 2020). Local inflammation involves the production of prostaglandins by inducible cyclooxygenase 2 (COX2) and the recruitment and activation of effector cells, such as polymorphonuclear neutrophils. This contributes to the development of pain and fever that occur during infection, which is the main reason why NSAIDs are so commonly used in infections. Alternatively, COX2 also has a pivotal role in the resolution of inflammation, thereby preventing self-inflicted damage from the immune response (Levy et al. 2001).

NSAIDs work by inhibiting the activity of cyclooxygenase enzymes (COX1 or COX2), thereby blocking the production of prostaglandins that are involved in the recruitment and activation of effector cells, such as polymorphonuclear neutrophils (PMNs). Given the dual role of COX2 in the inflammatory response, such as amplifying the initial acute phase and then promoting resolution, the question arises whether taking NSAIDs during SARS-CoV-2 infection could improve or complicate the course of the disease. The question becomes particularly important because of the two-phase immune response to SARS-CoV-2 infection: the first immune defense-based protective phase and the second inflammation-driven damaging phase leading to acute respiratory distress syndrome (ARDS), respiratory failure, and subsequent fatality (Shi et al. 2020). Due to the current novelty of COVID-19, data concerning the influence of NSAID use on COVID-19 outcomes remain needed.

A retrospective study of critically ill 2009 H1N1 influenza pandemic patients did not show any evidence that NSAID use affects mortality (Epperly et al. 2016). Following the SARS outbreak caused by SARS-CoV, investigators discovered that the NSAID indomethacin had direct antiviral activity against SARS-CoV via blocking viral RNA synthesis independent of COX inhibition (Amici et al. 2006). Furthermore, antiviral activity has been confirmed in vivo in dogs infected with canine coronavirus (Amici et al. 2006), but no human studies have been performed to confirm a positive effect for indomethacin treatment on SARS-CoV or SARS-CoV-2 infections. Notably, observational studies suggest an association between pre-hospital NSAID exposure and a protracted and complicated course of pneumonia (Voiriot et al. 2019). A survey by the French National Agency of Medicine and Health Products Safety revealed that infections caused by varicella and some bacterial infections could be aggravated by the NSAIDs ibuprofen and ketoprofen. Based on this report, the Safety Committee of the European Medicines Agency recently initiated a review of these drugs. The mechanisms by which NSAID use may lead to a complicated course of pneumonia are uncertain. It could be assumed that the intake of NSAIDs suppresses the main symptoms of inflammation, such as fever and pain, and therefore delays the timely detection of pneumonia and the subsequent start of appropriate therapy (Voiriot et al. 2019). Accordingly, most NSAID drug labels warn that these drugs could mask the signs or symptoms of infection. Another mechanism could be the immunological effects of NSAIDs, such as the impaired resolution of inflammation, thus resulting in prolonged disease (Voiriot et al. 2019).

## Conclusion and recommendations

In summary, there is currently no scientific evidence establishing a clear link between ARBs, ACE inhibitors, or ibuprofen and the worsening of COVID-19. In contrast, the experimental data support the idea that ACE2 not only serves as an entry receptor for SARS-CoV-2 but also protects the lungs from acute injury. RAS inhibitors may therefore even be beneficial in COVID-19 infection.

Available information to date does not support interruptions or changes in ongoing chronic treatments with ibuprofen or RAS blockers. Recommendations are as follows:

- Based on the currently available evidence, effective treatment with RAS blockers should not be discontinued or switched. The relatively hypothetical risk of worsening COVID-19 does not justify discontinuing or switching ARBs or ACE inhibitors due to the specific risk of destabilizing blood pressure control, stroke, heart attack, or worsening heart failure (van der Wardt et al. 2017; Halliday et al. 2019). Insufficient control of concomitant diseases caused by drug discontinuation could make patients more susceptible to SARS-CoV-2 infection and complicate the course of disease.
- Currently, there is insufficient evidence to recommend patients who take ibuprofen for medically indicated reasons to change their anti-inflammatory medication in light of the COVID-19 pandemic. NSAIDs should be used at the lowest effective dose for the shortest possible period. The choice of drug to treat fever or pain in COVID-19 should be based on a benefit-risk assessment for known side effects (e.g., kidney damage, gastrointestinal ulceration) and the respective treatment guidelines that in most cases recommend paracetamol as the first treatment option for fever or pain associated with infections.
